# Embedding indigenous and local knowledges with one health: a roadmap for convergence and synergies

**DOI:** 10.3389/fvets.2026.1819522

**Published:** 2026-08-13

**Authors:** Julie Garnier, Bernardo Couto Soares, Indira Khurana, Elizabeth Kola, Dominique Ouédraogo, Baldomero Molina-Flores, Juan Lubroth, Brigitte Bagnol, Daniel Kobei, Vincent Brioudes, Julia Hlukhonets, Akul Gupta, Rahul Narang, Zanan Coulibaly, Sisay Getachew, Raktima Mukhopadhyay

**Affiliations:** 1 Odyssey Conservation Trust, Bakewell, United Kingdom; 2 Knowledge, Technology and Innovation (KTI), Wageningen University & Research (WUR), Wageningen, Netherlands; 3 Tarun Bharat Sangh, Bheekampura, India; 4 School of Biology and Environmental Sciences, University of Mpumalanga, Nelspruit, South Africa; 5 Centre Universitaire de Ziniaré, Université Joseph KI-ZERBO, Ouagadougou, Burkina Faso; 6 Ibero-American Ethnoveterinary Network (RIEV) and Cordoba University, Cordoba, Spain; 7 Lubroth One Health Consultancies, Rieti, Italy; 8 Cummings School of Veterinary Medicine, Tufts University, Medford, MA, United States; 9 Ogiek People’s Development Program, Egerton, Kenya; 10 ENSV-FVI, VetAgro Sup, Marcy L’Etoile, France; 11 National University of Life and Environmental Sciences of Ukraine, Kyiv, Ukraine; 12 A.T. Still University - School of Osteopathic Medicine, Mesa, AZ, United States; 13 All India Institute of Medical Sciences Bibinagar, Hyderabad, Telangana, India; 14 Direction des Services Vétérinaires et du Bien-être Animal, Abidjan, Côte d'Ivoire; 15 Animal Health and Veterinary Public Health, Livestock and Fisheries Sector, Ministry of Agriculture, Addis Abebba, Ethiopia; 16 Indian Institute of Bio Social Research and Development, Kolkata, West Bengal, India

**Keywords:** co-creation, indigenous and local knowledge, justice, knowledge systems, one health, synergies

## Abstract

Establishing synergies between Indigenous and Local Knowledge (ILK) within One Health frameworks is crucial when addressing the intertwined crises of climate change, biodiversity loss, pollution, health, and widening social inequalities. Recognizing that different epistemologies, values and practices of different knowledge systems can equally contribute to One Health initiatives is essential to ensure that they are grounded in equity, respect, reciprocity and justice and that they can fulfil their objective of holistic health and well-being of all. Our objective is to highlight the value of building such synergies by drawing on cases studies that demonstrate the critical importance of indigenous and local knowledge and practices––beyond a purely biomedical approach––for strengthening resilience. We also develop a set of practical and policy-relevant recommendations to promote transdisciplinary collaboration between One Health and ILK actors. We propose advancing synergies and convergences across knowledge systems through the co-creation of transformative learning, governance reform, legal recognition, and knowledge co-production.

**Positionality:**

This article does not seek to speak on behalf of indigenous communities or local knowledge holders. It was co-developed by members of the International Working Group on Education and Communication of the One Sustainable Health for All Forum, most of whom are experienced practitioners engaged in community-led initiatives across different One Health domains around the world. As such, this paper is intended as a practice-oriented contribution to the One Health community, aimed at supporting more equitable, collaborative and impactful community-centred One Health initiatives.

## 1 Introduction

The convergence of unprecedented and interconnected crises––climate change, biodiversity loss, pollution, emerging infectious diseases and endemic diseases burden, and deepening inequities –now threatens the health and well-being of people and of the natural systems that sustain life on Earth. These crises, rooted in current systems of production and consumption and associated with resource extraction powered by fossil energy, food systems intensification, population growth, and technological innovation, urgently call for systemic solutions (1, 2). The systemic One Health approach recognises the interconnectedness between humans, animals, plants and the environment and is well suited to address complex challenges across multiple scale, but it must integrate the needs, values and perspectives of different stakeholders and knowledge systems (3–5).

Developing integrated One Health and Planetary Health frameworks with shared governance systems which fully value Indigenous and Local Knowledge systems is increasingly emphasised (6–9). Gender-responsive and rights-based One Health approaches highlight the need to align equity, inclusion, and reciprocity with indigenous and local communities (4, 10, 11). However, building equitable partnerships between stakeholders with different epistemological and ontological positions, such as One Health practitioners and indigenous knowledge holders, remains challenging, as Western scientific thinking and the biomedical paradigm continue to dominate (3, 12–15).

Indigenous and local communities hold deep knowledge of how to live in balance with nature—local^1^ knowledge rooted in observation, intergenerational transmission, spirituality, and reciprocal relationships with the land and living beings (9, 23). Their worldview, which may diverge from the human-animal-environment triad (16), integrate spiritual, mental, social, and physical well-being and health of all living forms, reflecting a holistic understanding of health that One Health and Planetary Health are trying to converge towards (4, 9). One Health practitioners must therefore reframe existing knowledge hierarchies to collaborate across and learn from different ways of understanding and living with nature. This is not only a matter of justice and equity, but of mere survival: Indigenous People steward much of the world’s biodiversity and Indigenous and Local Knowledge (ILK)^2^ is critical for climate resilience and biodiversity conservation (4, 9, 17, 18).

## 2 Indigenous and local knowledge: diverse uses across one health domains

We identified a set of diverse, thematically relevant topics across the human, animal, and ecosystem health domains to show the broadness of relevance of ILK in One Health. A narrative review (19) allowed for a critical analysis of the literature on each topic and to identify experts engaged in community-based work on these issues. These experts were invited to first contribute case studies highlighting the importance of grounding their work in indigenous and local knowledge.

Case study 1: Community-led decentralised water conservation for dignified livelihoods in the Chambal badlands^3^ of Rajasthan, India (based on Khurana, (24)).

The Chambal badlands (comprising gullies and ravines) are a rugged landscape, with water scarcity, poor soil, lack of vegetation and dreaded dacoits^4^ who hid in the gullies and ravines. The topography is punishing.

Many youths turned to crime because farming was impossible. With no other livelihood opportunities available and no food on the table, they turned to violence to feed their families.

Over the past three decades, hundreds of dacoits gave up their weapons and took up farming. Farming gave them a life of dignity: they can now meet their needs and aspirations. The change from a violent to a non-violent way of life was possible due to their community-led rainwater conservation efforts, which recharged the groundwater, improved soil moisture, vegetation health, and healed the earth. Over the years, water conservation led to the revival of small rivers, indicating a balance between groundwater and surface water. Forests regenerated, and wildlife had water to drink. Ecosystems began to revive.

Villagers are now more climate resilient. Migratory labour returned to their farms and began employing labour from other villages. As greenery increased, families began to raise livestock, since fodder was now easily available. Diets became more nutritious, and cash-in-hand increased, especially for women. Overall health and education improved. Aspirations began to be met: ramshackle dwellings were replaced by permanent structures, the purchase of vehicles increased and decentralised, more equitable and circular economies began to grow, as small shops came up to cater to local demand.

This work, spread across several small river basins, demonstrates the impact of capturing the humble raindrop, and of small, low-cost water conservation structures, constructed at appropriate sites, keeping in mind the local topography and the requirements of the villagers.

The use of local knowledge and wisdom in finding equitable solutions for water security was fundamental to the environmental, social and economic benefits that accrued, leading to villagers living with dignity, peace and security. This dramatic change has been possible due to the sustained community facilitation efforts of a civil society organisation, Tarun Bharat Sangh.

Case study 2: How safe, accessible drinking water changed women’s lives in Mewat district, India (based on Khurana, (24)).

Water is integral to achieving the vision of ‘One Health,’ and the Sustainable Development Goals (SDGs). Water nurtures life in all its forms, and thereby ecosystems. Climate change has deepened water insecurity, given that 90 per cent of all climate-related disasters are water-related and are now exacerbated by global warming. Evidence points to a myriad of ways in which women and girls are more affected by these disasters, which impact their health, safety and the health of the newborn.

Access to drinking water becomes a daily challenge, especially for women. To address the drinking water shortage faced by rural women in the Mewat district of Rajasthan, where summer temperatures are often beyond a life-threatening 45 °C, the NGO Tarun Bharat Sangh supported the construction of 100 household level 17,000-litre capacity drinking water tanks. These tanks are managed by the family members and, when empty, are refilled again with safe drinking water through tanker water supply.

Change is visible and palpable, especially for the women. Their health has improved, and they now have more time for themselves. Girl’s attendance in schools has risen. Agricultural activities improved and expanded due to the water conservation activities. Livestock rearing increased. Vegetable gardens sprang up and food plates displayed variety and colour, indicative of improved nutrition. The financial situation has improved and has become more stable.

*“My husband and I work as labour. I would need to spend 2–3 h to get water. Now that water is available at my home, I have enough time to work. My children are going to school. I have taken up stitching and now manage the accounts of my self-help group. I tend to my vegetable garden that provides for my kitchen,” s*hares Arzina, from Pathkori village.

Miskina, also from Pathkori, is the sole breadwinner in her family. She looks after her ailing husband and her children and takes care of their education. Her only source of income is the tailoring work that she does. Before, she was unable to devote much time to this because of the lack of water:

*“We would sometimes need to go for water in the middle of the night. People who owned the source would not let us fill water and would often break our containers. Thankfully, life has changed now.”*

The women are now able to engage in multiple business activities from their homes. They continue to labour in the fields, but also undertake tailoring jobs, and keep goats and buffaloes. Now milk and a good diet are available for the family, including for the women themselves.

**Figure 1:**
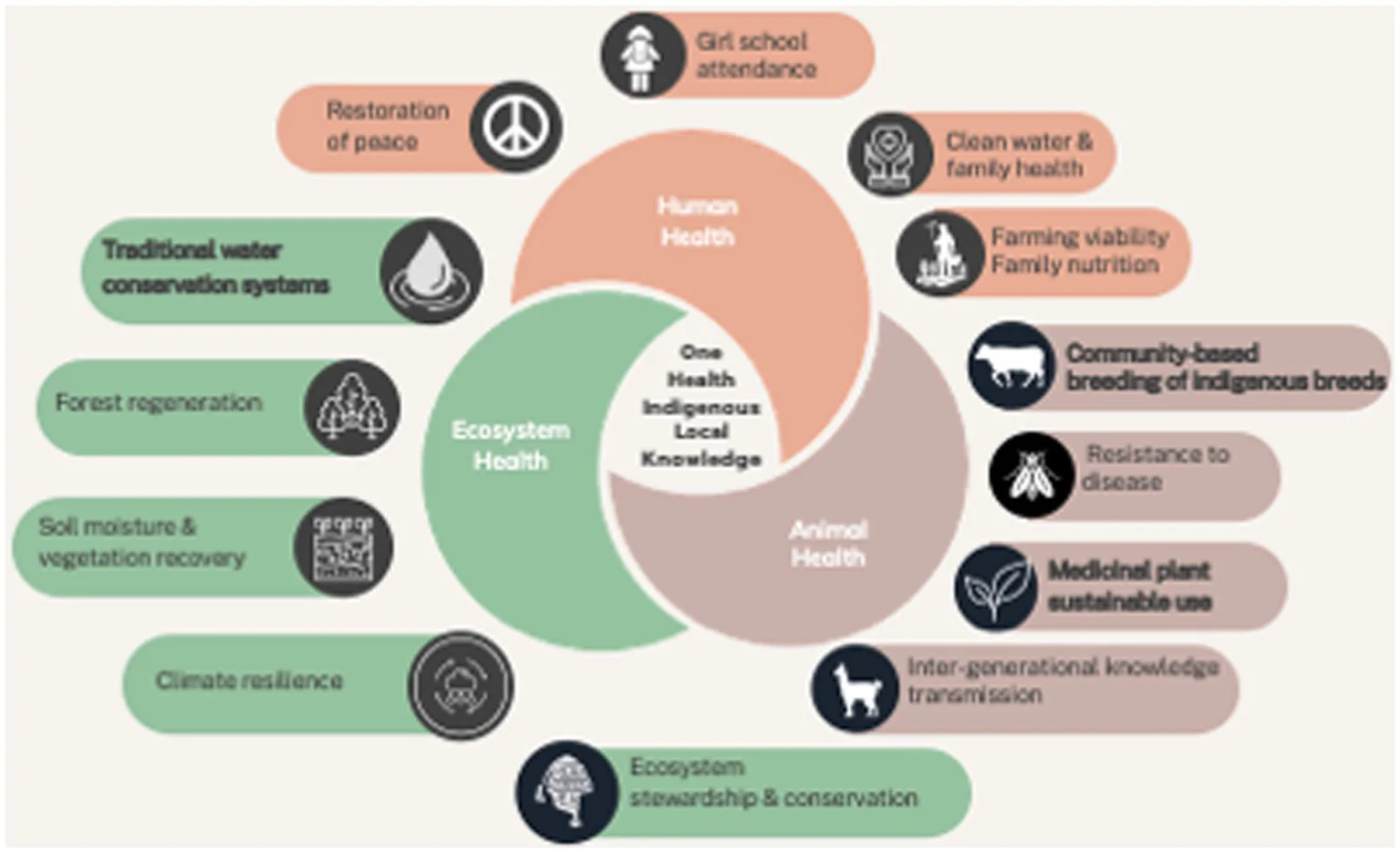
Indigenous and Local Knowledge contributions across the One Health domains, illustrated in the case studies. Bold labels indicate ILK entry points – the traditional practices that ground each case study. This figure is illustrative and not exhaustive.

Mehram from Bhoond engages in agricultural activities and livestock rearing, both of which are water dependent. Prior to this work, she and her entire family would roam around in their quest for water. While the world slept, they would be desperately searching for water. She shares:

*“We had to time our quest just right: There should be electricity and the people should be willing to let us take some water (Figure 1). Sometimes our containers would be broken and we would be insulted. There was just no dignity. These tanks have given us back our dignity and our livelihood.”*

Numbers speak:

A study undertaken in this area reinforces, reassures and reinforces existing commitment:

1. At least 80 per cent of women now have relief from back pain.
2. There has been a 60 per cent decline in diseases such as cholera.
3. There has been a 20 per cent increase in girls’ attendance at formal education.
4. At least 60 per cent of the families are now engaged in income-generating activities and small businesses by utilising the time they now have.

Case study 3: Strengthening Aymara ethnoveterinary knowledge for camelid health in the Chilean Andes (based on Manzano-Rada and Molina-Flores, (25)).

Llama and alpaca production is an ancestral practice linked to the way of life of the Aymara indigenous people of northern Chile. But this knowledge is at risk of disappearing due to the lack of generational renewal. Livestock policies have also contributed to its displacement and replacement with “modern” technical knowledge. A clear example is the heavy reliance of producers on conventional veterinary medicine rather than ethnoveterinary medicine.

Nevertheless, there is an increasing interest in rescuing, disseminating and strengthening Aymara ethnoveterinary practices, and several studies and initiatives are under way. Herders report a reduction in the effectiveness of veterinary drugs against ectoparasites and endoparasites and gastrointestinal infections, the main diseases affecting camelid production. Aymara ethnoveterinary practices include traditional treatments for these infections, as well as for other conditions, combining knowledge of the therapeutic potential of plant species with the preparation and administration of treatments, within a belief system closely linked to the Aymara worldview.

It is worth noting that the broad terminology (ethnosemantics and ethnotaxonomy) used to designate diseases, clinical signs, and remedies, may be relevant to the public veterinary service activities. This justifies the need to further investigate Aymara ethnoveterinary knowledge, enabling the development of collaborative mechanisms with the scientific community to highlight the relevance of ancestral knowledge in the context of adaptation to socioeconomic, cultural, and environmental changes. This knowledge will provide further background on the Aymara people’s way of life for the development of guidelines or criteria for government institutions to manage agricultural and cultural heritage, thereby promoting sustainable development around camelid production and contributing to the reduction of depopulation in the rural Andean region of northern Chile.

Case Study 4. Ethnoveterinary knowledge for sustainable rural development in Chiapas, Mexico (based on Gutiérrez and Molina-Flores, (26)).

While traditional agroecological practices have been successfully promoted in interventions focused on sustainable rural development in Chiapas’ indigenous communities, Mexico, the promotion of these practices in livestock farming—collectively referred to as ethnoveterinary medicine—has not received the same attention, even though they are mutually dependent and complementary. This ancestral knowledge regarding the prevention and treatment of animal diseases, livestock management systems, and other related practices, represents an alternative for low-income people living far from urbanised areas. It does not seek to replace conventional veterinary medicine, but rather to support and complement it. Its value is immense as it is based on knowledge, practices, and beliefs that for centuries have provided these communities with food, raw materials, and cultural and symbolic uses. These traditions have been handed down orally from generation to generation and are at risk of being lost in the near future as they are not recorded.

Various historical and contemporary factors have generated transformations in traditional animal production, triggering the decline, loss, and even hybridisation of ethnoveterinary knowledge. Despite this, ethnoveterinary continues to be a common practice that, today, is also emerging as an alternative for sustainable livestock production that is culturally appropriate and respectful of nature. Using participatory research techniques and supported by bibliographic documentation, several initiatives have been and are being implemented to rescue, validate, and implement ethnoveterinary knowledge and practices within Chiapas’ indigenous communities. These include the development of ethnoveterinary products, along with the search for a niche market, as well as users’ manuals that can help foster greater appropriation, dissemination, and recognition of these alternative practices, especially among young people, thus revaluing the peasant and indigenous knowledge of southeastern Mexico.

Case Study 5: Survey on the indigenous knowledge of medicinal plants in rural area (South Africa) (Kola, manuscript under preparation).

In South Africa the use of indigenous medicinal plants is common, especially in rural areas. However, their use is not well scientifically documented, and most people still have doubts as to their effectiveness. Their use is very important to rural communities because it saves money that would be used otherwise to buy western medicines and they are easy to access and prepare.

The study aimed to investigate the indigenous medicinal uses of *Ziziphus mucronata*––known as buffalo thorn––in Mphambo village, Limpopo Province, South Africa. A survey was conducted with 50 participants using purposive sampling and semi-structured face-to-face interviews. Ethnobotanical indices such as Relative Frequency of citation (RFC) and Use-Value (UV) were used for data analysis.

A total of 18 sub-categories of diseases were recorded and grouped into seven categories of diseases and one miscellaneous use category. Arthritis was the most dominant disease mentioned by participants in the study, with the highest RFC value of 0.22. In terms of UV, the pain and inflammation category had the highest value (0.36). Regarding plant parts usage, leaves (35%) were the most frequently used. Maceration (44%) and decoction (33%) were the main preparation methods, and plant remedies were mainly administered orally (68%).

*Ziziphus mucronata* therefore has a diversity of medicinal uses. The study also revealed that people in Mphambo village possess indigenous knowledge, although it is at risk of being eroded. Therefore, it is important to document indigenous knowledge of *Z. mucronata,* as its decline threatens the sustainable use and long-term survival of the plant. Documenting indigenous and local knowledge can support the development of effective conservation strategies by ensuring the plant’s presence in local ecosystems and its availability for future generations.

Case study 6: Community-based breeding programmes of the locally adapted taurine Lobi cattle in Burkina Faso: evidence for the conservation of indigenous breeds through the integration of community needs and values (based on (20)).

West Africa is one of the regions of the world that hosts substantial populations of *Bos taurus* cattle. These taurine cattle predominantly inhabit humid and sub-humid zones where trypanosomosis is endemic and have consequently developed trypanotolerance.

The Lobi taurine cattle are the most important taurine breed in Burkina Faso and are mainly found in the southwestern regions of the country, which are highly infested by tsetse flies (*Glossina* spp. the vector for *Trypanosoma* spp. in Africa). This breed is traditionally kept by specific ethnic groups, notably the Lobi people, from whom it derives its name. For these communities, Lobi cattle represent more than a production asset as they constitute part of their cultural heritage and play essential socio-cultural roles, including their use in dowries, funerals, and ritual sacrifices.

Beyond their social and cultural significance, Lobi cattle exhibit strong adaptation to the local environment, being able to survive and produce under low-input conditions with minimal supplementation in feed and veterinary care. During the rainy season, animals are generally managed under grazing systems, whereas in the dry season, they rely largely on scavenging and natural vegetation. Their trypanotolerance allows them to maintain low levels of parasitaemia and remain largely asymptomatic. Consequently, the use of veterinary drugs is limited, with farmers relying mainly on medicinal plants for animal health management––26 medicinal plant species were identified as being used to treat nine major cattle diseases in this region, with foot-and-mouth disease and trypanosomiasis being the most frequently treated.

Despite their socio-cultural and ecological importance, Lobi cattle—like most West African taurine breeds—are currently threatened by genetic erosion and possible absorption through indiscriminate crossbreeding with zebu cattle. Due to their relatively small body size and lower market value compared to zebu, farmers increasingly practice crossbreeding to produce larger animals that retain some degree of trypanotolerance. If not properly managed, such practices could ultimately lead to the extinction of the pure Lobi breed.

In this context, community-based breeding programmes (CBBPs) have been implemented since 2016 with the dual objective of improving productivity while conserving Lobi cattle genetic resources, with farmers being actively involved through local selection committees responsible for ranking and validating preferred breeding bulls, for example. Stakeholders agreed that conserving locally adapted indigenous breeds requires improving their productive performance to increase farmers’ interest in maintaining them. Within these programmes, breeding animals are selected based not only on estimated breeding values for production traits but also on farmers’ preferences for specific phenotypic and functional attributes.

The objective of farmer participation in the selection process is to ensure that selected animals meet their expectations and are effectively adopted. Results from successive selection events indicate that, while favouring higher-performing animals, farmers continue to highly value phenotypic appearance and culturally important traits.

After 8 years of implementation, the results of the CBBPs are encouraging. These findings demonstrate that community-based breeding approaches, by integrating farmers’ needs, preferences, and knowledge, can effectively support both genetic improvement and the long-term conservation of indigenous cattle breeds, in line with a One Health perspective.

The diversity of case studies presented above illustrates how ILK is being used to improve the health of humans, animals and ecosystems with many other benefits. In Rajasthan, ILK contributes directly to food and water security with cascading benefits for human health, women empowerment and ecological restoration. The revival of rainwater harvesting practices in Chambal badlands has helped to regenerate soils and vegetation, making farming viable again. These changes had multiple cascading effects on food security, human dignity and peace. In Mewat district, drinking water conservation had an impact on women’s health and education, water-borne diseases and household income.

In Mphambo village, Limpopo region in South Africa and the Andes in Northern Chile, local ethnobotanical knowledge exemplifies how cultural frameworks and ecological knowledge come together to support livestock health while conserving biodiversity, through traditions that limit over-harvesting and preserve ecosystems. Similarly, in the south-western regions of Burkina Faso, community-led breeding of Lobi cattle illustrates how local knowledge and co-creation of programmes supports the conservation of disease-resistant and locally adapted breeds.

Taken together, these six case studies show how ILK is inherently suited to address challenges in complex adaptive systems, being grounded in long-term observation, feedback-responsive practices, community learning, and relational worldviews that emphasise interdependence, adaptability, and resilience.

## 3 A roadmap towards advancing synergies and convergence

Between August and December 2024, we conducted three interdisciplinary workshops with members of our international working group and case studies contributors. We aimed to co-develop a shared set of principles that shaped a roadmap towards promoting synergies between One Health (OH) practice, policies and ILK, based on the guiding question: How do Western One Health scientists and practitioners engage, or fail to engage, with different knowledge systems? We used participatory outcome mapping to guide the process, which is recognised for mapping social change in complex adaptive systems (21).

Our stepwise approach included:

- Identifying priority objectives for creating synergies between ILK and OH;
- Exploring structural and epistemic barriers to achieving these objectives;
- Mapping necessary shifts in practice, policy, and mindset; and,
- Developing concrete, context-specific strategies and actions.

## 4 Discussion

### 4.1 Case studies on interactions between ILK and OH

### 4.2 Towards synergies between indigenous and local knowledge and one health

The participatory workshops (Table 1) reinforce a pattern already well documented in the literature: the current design and operationalisation of OH mostly fail to fully recognise, value, or integrate ILK. Addressing this requires a deliberate shift in mindset, practice, and policy, grounded in inclusive collaborations and the co-creation of projects. In the following subheadings we discuss our workshops’ results, connect insights to broader scholarship and propose further actions.

1. Develop frameworks that respect differences

**Table 1:** Workshop-derived roadmap for advancing synergies between indigenous and local knowledge and one health.

Focus area	Objectives	Obstacles	Necessary changes and expected outcomes	Activities
Core values	Developing frameworks that respect differences between One Health (OH) knowledge and ILK	One Health remains an anthropocentric perspective, biomedical focused and single world approach Weak representation of ILK in OH Extractive consumerism is predominant	Recognise diverse visions of well-being and ways of connecting with nature Provide greater visibility for local and indigenous worldview in OH frameworks Shift towards a regenerative economy	Develop methodologies and tools to facilitate collaboration between ILK scholars and stakeholders with OH practitioners Promote regenerative economy models inspired by local and indigenous stewardship
Community engagement	Co-CREATING local initiatives involving ILK stakeholders	Weak intergenerational transmission of ILK Limited recognition of ILK and local participation in decision-making	Strengthen inter-generational transmission of ILK Revitalise cultural practices Increase indigenous people’s agency in OH programmes	Support inter-generational education programmes Identify and promote cultural practices related to OH domains Foster economic sustainability grounded in ILK
Training	STRENGTHENING the capacity-building of trainers INTEGRATING ILK at all levels of education	Lack of training in social sciences for One Health practitioners Disconnect between scientists and field realities	Enhance intercultural and GESI (Gender Equity and Social Inclusion) competencies among One Health practitioners Promote collaboration between scholars and local communities	Integrate social sciences and ILK into One Health training Encourage immersive field missions and experiential learning for OH students and scientists Develop partnerships with indigenous communities and local institutions for the co-delivery of education programmes
Communication	Promoting knowledge sharing between scientists, policymakers and local communities	Poor communication between diverse stakeholders Limited access to knowledge-sharing platforms.	Ground OH policies and interventions in local contexts Improve accessibility and inclusion of communication platforms and tools	Establish regional knowledge sharing hubs (radio programmes, storytelling, etc.) Co-design culturally relevant communication tools Identify and promote ILK within media and with OH practitioners
Advocacy	Advocating for legal frameworks to protect and include ILK in policy development Promoting the multiple values of ILK Elevating local and indigenous representation in decision-making	Lack of legal protections against bio-piracy Absence of economic data to demonstrate ILK values	Ensure recognition of ILK and IP rights in legal and policy frameworks Increase political will to protect ILK from misappropriation and IP’s rights Strengthen community participation in governance processes	Promote legal protections and policies that recognise ILK’s cultural and economic contributions Conduct policy advocacy campaigns and promote legal frameworks like the Nagoya Protocol
Research and documentation	Collaborating with scientists from local communities	Lack of scientific validation of ILK Loss of ILK due to environmental degradation and rural to urban migration	Co-develop collaborative research frameworks with Indigenous and Local Communities Co-produce knowledge that respects ILK epistemologies Make ILK databases accessible	Establish guidelines for ILK-inclusive research Co-author publications with indigenous scientists Develop multi-lingual and culturally appropriate ILK databases
Policy and governance	Fostering collaborative approaches for One Health policymaking Ensuring democratic processes, Integrating ilk into biodiversity and health management	Dominance of top-down policy approaches Exclusion of Indigenous voices from decision-making	Establish inclusive OH governance structures Fully integrate ILK in One Health action plans Shift toward bottom-up decision-making OH models	Facilitate indigenous-led policy dialogues on OH-related domains Develop OH action plans that embed ILK into national OH strategies

Knowledge plurality becomes critical, for recognising that ILK cannot be evaluated by the same standards and criteria as Western science demands.^5^ Practical actions include co-creating regular spaces for dialogue between ILK stakeholders and OH actors, developing frameworks that incorporate non-Western concepts of health, and learning from ILK models of stewardship and regeneration. Ludwig and El-Hani (22) recommended partial overlaps for a reflective approach that respect the autonomy and self-determination of local communities. Transdisciplinary collaboration requires careful attention for being able to craft compromises and find ways of accommodating goals that may initially diverge but can evolve to establish a common vision that respects different worldviews and promote synergies.

1. Co-create local initiatives involving ILK stakeholders

One of the clearest concerns emerging from the workshops was the weakening of ILK transmission due to rural-to-urban migration, youth disengagement, and the undervaluation of indigenous and local knowledge systems. Addressing this requires investment in pedagogies that affirm ILK. This includes supporting youth apprenticeships and mentorships, developing local seed banks and herbal gardens, promoting biocontrol methods of managing pests, soil health management and water conservation, ethnobotanical training, and participatory animal health and breeding programmes as a few examples. Strengthening ILK transmission not only preserves knowledge—it enhances the adaptive capacity of all by ensuring that communities can respond to environmental and health shifts with context-specific strategies. These initiatives act as stabilising feedback loops in complex systems, where knowledge, identity, and sustainability reinforce one another.

1. Reform One Health education to co-creating trainings with ILK stakeholders

We found that many One Health practitioners lack training, and possibly interest, in intercultural engagement, social sciences, and ethics. This gap undermines their ability to work effectively with ILK stakeholders in a truly collaborative and just manner. Reforming OH curricula to include decolonial scholarship, GESI^6^ studies, and political ecology would prepare practitioners to engage in collaborative, context-sensitive problem-solving. Co-creating training modules, field immersion programmes, and partnerships with ILK stakeholders can help equip future professionals with the competencies needed for transdisciplinary work. These educational reforms represent high-leverage interventions in OH system thinking and shaping mindsets early in professional trajectories.

1. Develop culturally grounded, and inclusive communication platforms

Co-creating accessible and culturally relevant communication tools for facilitating trust and knowledge exchange is paramount. This includes investing in communication tools that promote ILK traditions—such as radio, storytelling, and participatory video—as well as recognising communicators and artists as essential voices and disseminators of values and good practices. Building regional knowledge-sharing hubs can also create feedback loops between science, policy, finance, and communities. These platforms help decentralise communication and promote mutual understanding, especially in contexts where technical language and institutional jargon can represent barriers to inclusion.

1. Legally recognise and protect ILK and communities rights

Legal and regulatory frameworks must move beyond symbolic inclusion and toward substantive protections for ILK and the rights of its holders. Workshop participants emphasised the need for policies that reflect the economic, cultural, and ecological value of ILK. National legislation should align with the Nagoya Protocol^7^, the UN Declaration on the Rights of Indigenous Peoples^8^, and local customary laws. Indigenous peoples need to be included in governance structures and processes, and their contributions in biodiversity and health strategies and in all domains need to be promoted. These frameworks anchor ILK within a broader system of resilience by securing the institutional space needed to ensure its continuity and legitimacy. Strengthening country’s laws for involving indigenous communities in governance mechanism, restoring their land rights, and providing them with the statutory powers needed for managing biodiversity, while negotiating for Access and Benefit Sharing^9^ arrangements and patenting their knowledge following the CBD guidelines are also paramount.

1. Support co-creation and ethical research partnerships with ILK stakeholders and communities

Research systems often support epistemic injustice, where ILK is either extracted without benefit or excluded. A shift toward co-creation—where local communities are equal partners in framing questions, generating knowledge, and interpreting results—is essential. Participatory methods, co-authorship, and ethical review processes that respect ILK data sovereignty are critical first steps. Funding should be directed to long-term partnerships that support mutual capacity building and benefit-sharing. Co-designed research not only produces more relevant findings; it creates adaptive learning cycles that benefit both One Health and ILK (8, 9).

1. Establish inclusive and bottom-up governance structures

Top-down approaches remain a major obstacle to ILK inclusion. Our workshops revealed the need for more democratic, participatory decision-making processes in OH policy at local, national, and international levels. Mechanisms for ILK stakeholders —such as permanent advisory councils, co-management boards, and Indigenous-led assessments—should be institutionalised in OH strategies. Governance structures should reflect the plurality of cultural frameworks and allow policies to be shaped by all stakeholders involved. By decentralising governance and recognising the legitimacy of ILK, One Health projects can become more adaptive, equitable, sustainable and impactful.

## 5 Conclusion

Despite everlasting rhetoric about inclusion, Indigenous and Local Knowledge systems remains marginal in most operational One Health strategies. Without addressing structural barriers—such as epistemic hierarchies, legal invisibility, and exclusion from governance processes—this gap will persist. The case studies and workshop findings presented demonstrate that ILK provides grounded, culturally meaningful, and ecologically sound pathways to tackling current polycrisis. These knowledge systems are not remnants of the past—they are living, evolving, and deeply connected to the territories and communities that steward some of the world’s most biodiverse and climate-vulnerable landscapes.

We call on governments, donors, academic institutions, and civil society organisations to take bold steps: reimagine governance to include ILK stakeholders; reform education to embrace critical scholarship and co-creating trainings with local and indigenous communities; protect Indigenous rights through legal mechanisms; and invest in co-creation processes that respect, valorise and amplify ILK epistemologies.

## Data Availability

The original contributions presented in the study are included in the article/supplementary material, further inquiries can be directed to the corresponding authors.

## Footnotes

1 We use the word “local” to refer to a knowledge that is situated in time and place and which is specific to a social group and or a region. “Traditional” is often understood as a knowledge or a practice passed on from generation to generation in an unchanged pattern. Anthropological studies of these practices highlight their changing patterns under the influence of socio-economic and cultural factors such as education, contact with other knowledge, technology, etc.

2 We use the term ‘Indigenous and Local Knowledge’ or ILK to capture the immense diversity and complexity of knowledge associated with local communities Hill et al. (23). These communities, while not always self-identified as indigenous, hold knowledge systems and stewardship practices grounded in centuries of lived experience.

3 The Chambal Basin harbours unique badlands––rugged landforms shaped through climatic, tectonic and erosional processes- which exhibit considerable depth, high drainage density, and extensive ecological richness.

4 Dacoits are members of an armed gang of robbers.

5 As an example, there are overlaps in recognising certain animals or plants, but differences can arise in wider cosmological views, such as an animistic perspective on landscapes. The ways different knowledge systems consider their worlds may differ in their methods for generating and validating knowledge.

6 GESI studies analyse how different groups—especially those historically marginalised—experience policies, programs, and systems, with the goal that interventions are equitable, inclusive, and responsive to diverse needs. The studies (study design) consider the following intersecting factors: Age, Gender, Disability, Ethnicity and Indigenous identity, Socioeconomic status, Geographical (i.e., rural/urban/peri-urban), Sexual orientation and gender identity, Migration status.

7
https://www.cbd.int/abs/about

8
https://www.ohchr.org/en/indigenous-peoples/un-declaration-rights-indigenous-peoples

9
https://www.cbd.int/abs/infokit/brochure-en.pdf
